# Accelerated Shape Forming and Recovering, Induction, and Release of Adhesiveness of Conductive Carbon Nanotube/Epoxy Composites by Joule Heating

**DOI:** 10.3390/polym12051030

**Published:** 2020-05-01

**Authors:** Petr Slobodian, Pavel Riha, Robert Olejnik, Jiri Matyas

**Affiliations:** 1 Centre of Polymer Systems, University Institute, Tomas Bata University, Tr. T. Bati 5678, 760 01 Zlin, Czech Republic; olejnik@utb.cz (R.O.); matyas@utb.cz (J.M.); 2 The Czech Academy of Sciences, Institute of Hydrodynamics, Pod Patankou 5, 166 12 Prague 6, Czech Republic

**Keywords:** carbon nanotubes, epoxy, Joule heating, fast curing, accelerated forming, shape memory

## Abstract

The versatile properties of a nanopaper consisting of a porous network of multi-walled carbon nanotubes were applied to enhance the mechanical and electrical properties of a thermosetting epoxy polymer. The embedded nanopaper proved useful both in the monitoring of the curing process of the epoxy resin by the self-regulating Joule heating and in the supervising of tensile deformations of the composite by detecting changes in its electrical resistance. When heated by Joule heating above its glass transition temperature, the embedded carbon nanotube nanopaper accelerated not only the modelling of the composites into various shapes, but also the shape recovery process, wherein the stress in the nanopaper was released and the shape of the composite reverted to its original configuration. Lastly, in comparison with its respective epoxy adhesive, the internally heated electro-conductive carbon nanotube nanopaper/epoxy composite not only substantially shortened curing time while retaining comparable strength of the adhesive bonding of the steel surfaces, but also enabled a release of such bonds by repeated application of DC current.

## 1. Introduction

We recently used a nanopaper made of multi-walled carbon nanotubes (MWCNTs) to monitor infiltration of epoxy resin through a glass fiber textile and study the curing process and deformation of a glass fiber/epoxy composite [1]. The embedded MWCNT nanopaper offers further opportunities for technological applications. In this paper, we introduced curing of carbon nanotube/epoxy composites by Joule heating, and forming of the composites into various shapes. This was enabled by rapid tempering of the composite above its glass transition temperature by means of Joule heating, feasibility of repetition of this reheating and reshaping without losing the material properties, and efficient adhesive bonding of metals by these composites.

The idea of curing of carbon allotrope/epoxy composites by means of self-heating has proven successful in several composite arrangements. For instance, carbon fiber/epoxy composites can be cured by Joule heating. Such cured composites compare favorably with oven-cured ones, thanks to the lower energy consumption of the Joule heating process [2]. Similarly, epoxy resins with dispersed multiwall carbon nanotubes (MWCNTs) or graphene nanoplatelets can be cured well by Joule heating. Examples of practical applications of such curing processes are the use of an epoxy resin with MWCNTs as an adhesive to repair aerospace composite parts or soldering of an assembly of MWCNTs to a metallic surface [3]. Joule heating has been also used to cure an epoxy composite with an embedded single-wall carbon nanotube network and graphene nanoplates supported by glass fibers, which has been applied to deicing [4,5]. A self-regulating out-of-oven manufacturing of a fiber-reinforced epoxy by an embedded layer of graphene nanoplatelets is described in [6]. When compared to state-of-the-art out-of-autoclave oven curing, the curing by nanoplates consumes only 1% of the energy required for respective curing in the oven and has no negative effects on the mechanical performance and glass transition temperature of the final composite. Similarly, the curing by Joule heating of the graphene nanoplatelets/epoxy mixtures results in more compact composite structures with fewer micro-voids and enhanced electrical and mechanical properties than in the respective oven cured composites [7].

It has recently been shown that polypropylene with an embedded carbon fiber veil can be molded into a composite product by Joule heating, with properties comparable to that prepared by an oven heating process [8]. Similarly, since comparable bonding strengths of electrically cured and conventional adhesives has been achieved, Joule heating was successfully used to bond parts made of conductive or non-conductive materials with a MWCNT/epoxy composite [9,10] made of a conductive carbon black/epoxy-based shape memory polymer [11] or a carbon fiber mesh embedded in epoxy [12]. The controlled heating to achieve the desired bondline temperature for the out-of-autoclave adhesive bonding of carbon-fiber composite components has been tested in [13]. The control of the bondline temperature is arranged without an embedded sensor, which can cause a reduced bond strength. The conductive self-healing nanocomposites, which consists of multi-walled carbon nanotubes dispersed into crosslinked polyketons can repair emerged microcracks. The healing effect induced by electricity prolongs the service life of polymer nanocomposites and improves product performance [14]. The flexible piezoresistive sensors based on the RTV-silicone and milled carbon fibers change their resistance when an electric current is applied and Joule heating increases their temperature. The resistance change is caused by swelling of the polymer matrix, which reduces interconnections between the milled carbon fibers [15].

Different embedded reinforcing conductive structures in polymer matrices increase strength and add electrical properties to such polymer composites. These properties may be used for forming shapes of industrial products and parts or for an electrical-driven actuation, among others. The recent progress in the investigation of shape memory polymer composites with embedded carbon black, carbon nanotubes (CNTs), carbon nanofibers (CNFs), and graphene is reviewed in [16]. The carbon black distribution in a polymer matrix correlates with the shape recovery of the composite, which is triggered by applying voltage [17]. While uniform distribution of the spherical carbon black fillers diminishes the chance of their contacts, carbon black aggregates form bunches of microcircuits, which facilitate the shape recovery of such composites when applying a voltage. In addition, a shape memory polymer is employed to form a new composite with temperature and water sensing, as well as actuating capabilities. The shape memory function of this composite is characterized using a Joule heating-based activation method to understand shape recovery at different temperatures [18]. A low-voltage-driven transparent actuator, which is made from a polymer and single-layer carbon nanotube film composites, is used in variable-focus lens. The respective actuating mechanism depends on a large volume change of polymers when they are Joule-heated by electrical current [19]. 

The above examples show possible versatile uses of the various carbon allotrope structures embedded in epoxy polymers or in similar polymers. However, we think that the use of the carbon nanotube nanopaper embedded in the epoxy in various technological applications is still insufficient. In our previous paper [1], we described the monitoring of curing and deformation of a glass fiber reinforced epoxy by an integrated MWCNT nanopaper. Here, we showed the embedded MWCNT nanopaper in several further applications: induction of rapid Joule heating of the MWCNT nanopaper/epoxy composite, sped-up curing, facilitating of shape recovery of a modelled composite formation to its original configuration, and bonding of parts with shorter times and enabled release of such adhesive bonds by repeated application of DC current.

## 2. Materials and Methods

Purified MWCNTs produced by chemical vapor deposition of acetylene were supplied by Sun Nanotech Co. Ltd., Jiangxi, China. According to the supplier, the nanotube diameter was 10–30 nm, length 1–10 μm, purity ~ 90%, and volume resistivity 0.12 Ω cm. Further details about the nanotubes and results of the transmission electron microscope (TEM) analysis can be found in our previous papers [20,21].

The aqueous dispersion of MWCNTs (0.8 mg of nanotubes, 530 mL of water with a surfactant system based on a solution of 15.4 g sodium dodecyl sulphate as a surfactant agent and 8.5 mL of 1-pentanol as a co-surfactant agent) was prepared by sonication using the Dr Hielscher GmbH UP400St apparatus (ultrasonic horn S7, amplitude 88 μm, power density 300 W/cm^2^, frequency 24 kHz, (Hielscher Ultrasonics GmbH, Teltow, Germany) for 15 min at 50% power of the apparatus, 50% pulse mode, and temperature about 50 °C. NaOH aqueous solution was added to adjust pH to 10. 

To make an entangled MWCNT nanopaper (denoted as MWCNTnanopaper further on) from pristine nanotubes, the nanotubes were deposited on a porous polyurethane electrospun non-woven membrane by vacuum filtration. Two hundred and fifty mL of the homogenized MWCNT dispersion was filtered through a funnel 90 mm in diameter. Then, the resulting disk-shaped filtration cake was washed in situ several times with deionized water (at 65 °C) and afterwards with methanol. The filtering membrane was peeled off and dried between two filtration papers for 24 h at room temperature. The measured electrical conductivity of the MWCNT nanopaper was S = 11.97 S/cm.

The two-component epoxy resin Epox G 200 (Davex Chemical s.r.o., Prague, Czech Republic) is a transparent epoxy casting system with adjustable hardness and extended processing time. According to the supplier, the ratio of the epoxy components (A/B-hard/elastic) 100:50 yields a hard epoxy of hardness 79 Shore D, while the component ratio of 100:100 yields a flexible one (44 Shore A). The glass transition temperatures *T*_g_, which was determined by differential scanning calorimetry (DSC) analysis (DSC 1, Perkin Elmer), were 18.4, 35.1 and 58.1 °C for the component ratios of 100:100, 100:75, and 100:50, respectively. The chosen mixing epoxy component ratio of 100:75 enabled manipulation and shape forming of experimental epoxy samples above *T*_g_ (35.1 °C) manually. The curing time was 48 h at room temperature. The exothermic heat of the curing was determined by the DSC analysis as −275 J/g and peak temperature 140 °C at a heating rate 10 °C/min. The cured epoxy was elastic and flexible (without any damage at the bending over radius of 70 mm); its ultimate tensile strain was 3.5% and hardness 66 Shore D. 

The samples of MWCNT nanopaper/epoxy composite for the tests were fabricated in steps, which included at first the gluing of Cu strip electrodes to the opposite sides of a nanopaper plate. Then, the plate was put on a polytetrafluorethylene (PTFE) foil, filled with epoxy resin, and its surface covered by the PTFE foil again. To avoid wrinkling in the course of curing by inner Joule heating at a temperature around 100 °C for 6 min, the plate was kept under a pressure load of 300 kPa. The measured electrical conductivity of the resulting composite was S = 3.16 S/cm. The glass transition temperature of the epoxy matrix with the embedded nanopaper, determined as peak temperature, was 53.9 °C.

To illustrate the shape recovery of the bent strips of the MWCNTnanopaper/epoxy composite induced by Joule heating, two electrodes were attached lengthwise to the opposite sides of the strip (length 40 mm, width 20 mm). The electrodes were made of Cuprexit (a thin copper layer supported by a 0.22 mm thick fiberglass plate). The composite strip was stapled to the conductive copper layer of the Cuprexit electrodes at 60 °C, to which in turn were soldered conductive wires. Subsequently, the composite strip was warmed up by Joule heating to about 60 °C and molded by hand alongside an edge to form a right-angled shape. The composite strip was held in that shape by hand until the material cooled to below its *T*_g_ and thus retained the imposed shape. Thereafter, the voltage was reapplied, and the deformed composite strip straightened against gravity, regaining its original flat shape.

An identical composite strip was warmed in an oven to 60 °C and a part of the strip was reeled onto a glass rod. It was then held by hand in that shape until the material cooled below its *T*_g_ and thus retained the imposed shape. The straight portion of the partially coiled composite strip was attached by double-sided tape to a vertical side of a box and the coiled portion was left protruding above. Next, the misshapen composite strip was reheated in an oven to 60 °C and its coiled portion straightened against gravity, regaining its original flat shape.

Thermogravimetric analysis (TGA) of the epoxy and MWCNT nanopaper/epoxy composite samples was carried out using the thermogravimeter SETARAM SETSYS Evolution 1200 (METTLER TOLEDO, Prague, Czech Republic). The samples were examined under inert atmosphere of helium (5.5 purity, SIAD TP s.r.o., Prague, Czech Republic), which minimized the oxidation-dependent weight fraction loss of carbon nanotubes. Gas flow of 30 cm^3^/min at pressure of 101.325 kPa (i.e., 30 sccm) was set for all experiments. A platinum crucible was used to hold the sample, which weighted about 4 mg. The temperature was increased at a rate of 20 °C/min within the range from ambient room temperature to 1200 °C.

The structure of the MWCNT nanopaper and the cross-section of the nanopaper/epoxy composite were analyzed by a scanning electron microscope (SEM) (NOVA NanoSEM 450, FEI Co., Advex Instruments, Brno, Czech Republic). The surface temperature of the specimens was measured by means of a thermal camera (Flir E5-XT, FLIR Systems, Inc., Sally Gao, China), which was able to create a detailed temperature pattern. Sensor resistance was measured lengthwise by the two-point technique using the Multiplex datalogger 34980A (KEYSIGHT Technologies, Santa Rosa, CA, USA), which stored the readouts once per second. The DC power supply Metex AX 502 (AEMC Instruments, Dover, DE, USA) was used to power the Joule heating of composites. Tensile tests were carried out using the Testometric M350-5CT system (Testometric Co. Ltd., Rochdale, UK).

## 3. Results

**Figure 1:**
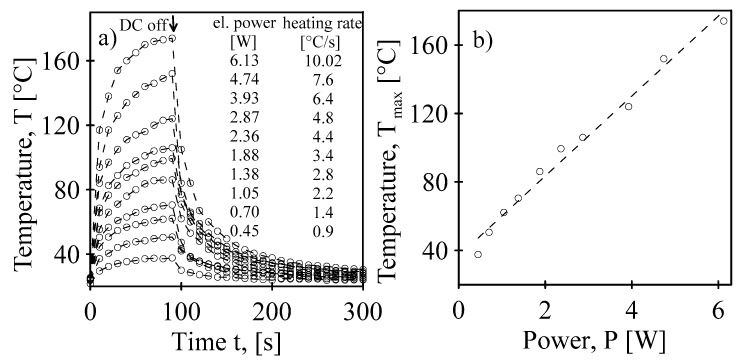
(**a**) Temperature progression of the MWCNT nanopaper through Joule heating at different electrical power rates and an initial heating rate. (**b**) The dependence of the reached maximal temperature on the applied DC electrical power through Joule heating.

The temperature progression induced by the imposed electric power on the MWCNT nanopaper and the dependence of the reached maximal temperature on the electric power are shown in Figure 1.

**Figure 2:**
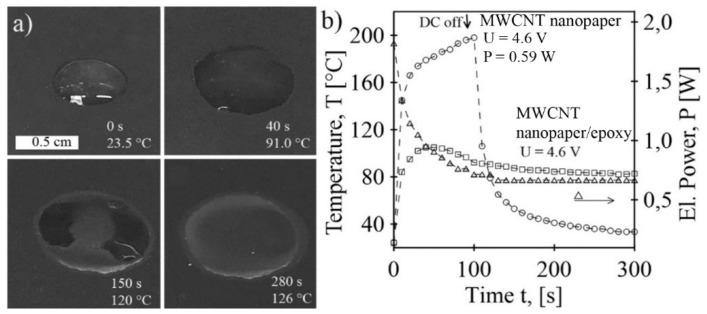
(**a**) Time-dependent curing of the epoxy resin droplet on the surface of the entangled MWCNT nanopaper by Joule heating at constant DC voltage. Respective times of curing and corresponding temperatures are indicated in the figure. (**b**) Temperature progression in the course of MWCNT nanopaper/epoxy curing by Joule heating at DC voltage 4.6 V is denoted by squares, the corresponding time dependence of the electrical power by triangles, and the time-dependence of the temperature during the MWCNT nanopaper heating at DC voltage of 4.6 V by circles.

The process of epoxy resin curing is shown in Figure 2a. The changing shades of grey in the photographs correspond to the advancing cross-linking of epoxy resin at the indicated times and temperatures. The temperature progression of the composite in Figure 2b indicated at first progressive resistive heating by DC voltage 4.6 V through inter-tube contacts, up to a temperature of 105 °C, which was reached in about 40 sec. The following temperature decrease was apparently a consequence of epoxy resin polymerization, which affected the number of inter-nanotube contacts and/or the contact resistance and resulted in a reduction of the applied power. On the other hand, when the same voltage was applied, the temperature of the MWCNT nanopaper continuously rose, thanks to the unaffected nanotube contacts until the temperature reached the arbitrarily chosen limit of 200 °C. 

The presence of MWCNTs affected the curing kinetics, which was obvious from the value of the glass transition temperatures of pure epoxy and of the MWCNTnanopaper/epoxy (100:75) composite. The measured *T_g_* as peaks by the DSC was 35.1 °C for the former and 53.9 °C for the latter. The peak shift to the higher temperature was similar to the effect of the decreasing of the ratio between the resin components A:B in favor of component A. The peak shift was not due to creation of the second phase of epoxy as the immobilized rigid amorphous fracture near the MWCNT surface [22]. Only one enthalpy relaxation peak was measured and enthalpy relaxation, as expressed by the term of enthalpy losses [23], was not changed by the MWCNT presence. The single enthalpy relaxation peak represents transition energy from the glassy state to the rubbery state and is not affected by the embedded MWCNTs [23].

Component A was an epoxy resin prepolymer (hydrogenated bisphenol A polymer with epichlorohydrin [CAS: 30583-72-3]). The prepolymer was prepared with sufficient excess of epichlorohydrin so the termination was by free oxirane rings capable to react chemically with amine groups. Component B (Trimethylolpropane tris[poly(propylene glycol), amine terminated] ether [CAS: 39423-51-3]) was a hardener or a curing agent containing three polymeric chains of poly(propylene glycol) terminated by amine groups. The poly(propylene glycol) chains themselves are highly movable, capable to rotate around a single chemical bond. The chains contain oxygen, which has a relatively low rotational barrier, and therefore the conformation of the chains changes easily under, e.g., an applied mechanical stress. The reaction of components A and B leads to the formation of a three-dimensional polymer network, resulting in a final epoxy matrix. At an excess of component B at the 100:100 ratio, some poly(propylene glycol) branches remain unbounded, by a lack of oxirane rings with a high molecular mobility potential, and the epoxy is elastic. With a lack of component B at the 100:50 ratio, the proportion of functional groups for crosslinking is more equimolecular. The epoxy contains less free unbounded poly(propylene glycol) branches, resulting in resistance against conformation changes, stiffness, and a higher *T_g_*. The epoxy curing in the presence of MWCNTs, which have oxygenated functional groups on their surface [24], increases epoxy matrix rigidity since the amine groups link to the carboxylic groups attached to the nanotubes, forming imide covalent bonds [25]. It was also confirmed independently that the curing of melamine-formaldehyde resin in the presence of the COOH-functionalized MWCNTs was affected by amine groups [26]. 

X-ray photoelectron spectroscopy (XPS) signals were measured to obtain information on functional groups attached to the nanotube surfaces [24]. The signals from MWCNTs were recorded by the Thermo Scientific K-Alpha XPS system (Thermo Fisher Scientific, UK) equipped with a micro-focused, monochromatic Al Kα X-ray source (1486.6 eV). The main binding energy peak (284.5 eV) in the XPS spectra of MWCNTs was assigned to the C1s–sp^2^, while the other ones were assigned to the respective oxygenated functional groups C–O (286.2 eV), C=O (287.1 eV), O–C=O (288.6–289 eV), and C1s–π–π^*^ (291.1–291.5 eV). 

**Figure 3:**
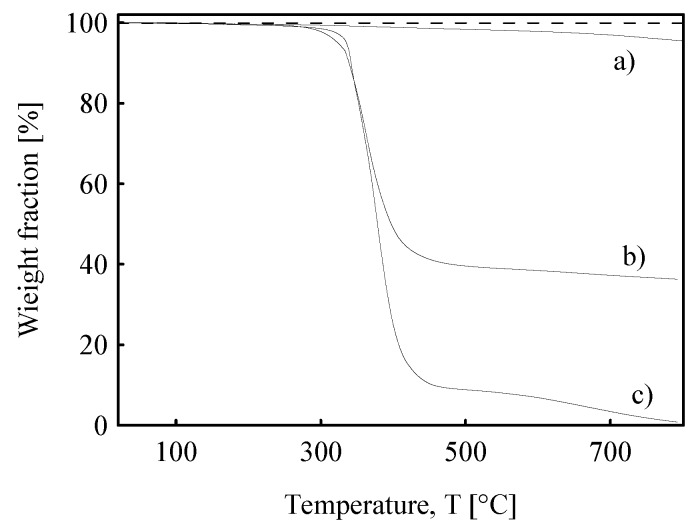
TGA curves of (**a**) the pure MWCNT nanopaper, (**b**) the MWCNT nanopaper/epoxy composite, and (**c**) the cured pure epoxy matrix.

A TGA analysis was performed on the MWCNT nanopaper, the MWCNT nanopaper/epoxy composite, and the cured epoxy to determine the weight fraction of MWCNTs, Figure 3. The TGA curves remained flat until the temperature reached about 300 °C when a loss of weight of the composite and the cured epoxy was observed owing to a decomposition of the epoxy. The weight percentages of the embedded MWCNTs in the composite after heating was (slightly) below 40 wt.%, which corresponded reasonably well to the weight fraction of the MWCNT nanopaper with the calculated porosity $\varphi =0.67=1-\rho_{nan}/\rho_{MWCNT}$, where $\rho_{nan}$ = 0.56 ± 0.03 g/cm^3^ denoted the measured apparent density of the nanopaper and $\rho_{MWCNT}$ = 1.7 g/cm^3^ the measured average density of nanotubes, which was close to the theoretical value of 1.8 g/cm^3^ [12].

**Figure 4:**
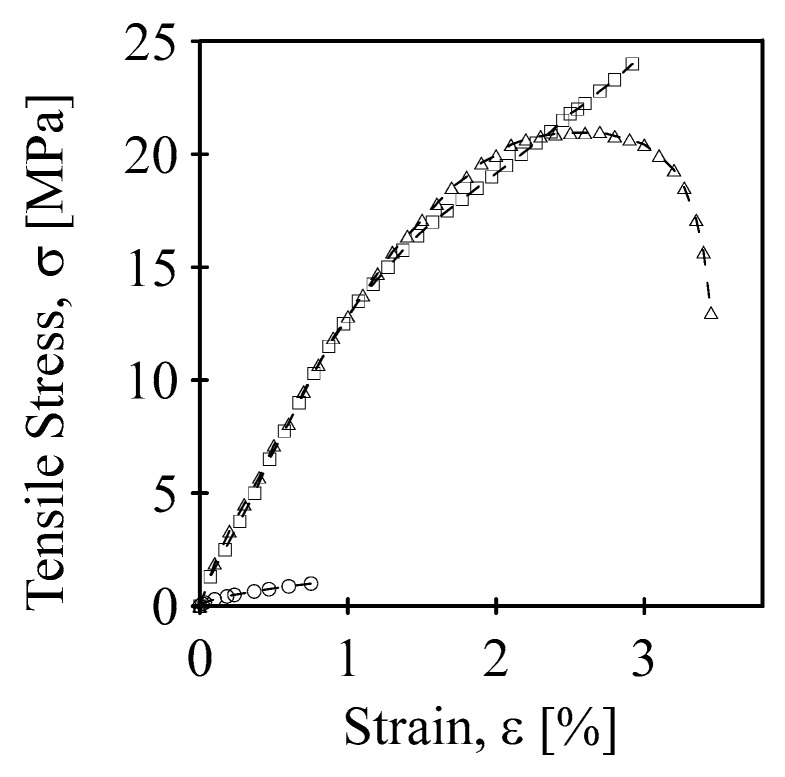
Tensile tests on the MWCNT nanopaper (circles), the MWCNT nanopaper/epoxy composite (squares), and the cured epoxy (triangles) at elongation rate of 5 mm/min.

A tensile test of the MWCNT nanopaper, the MWCNT nanopaper/epoxy composite, and the cured epoxy was carried out to determine their fracture tensile strength and strain. According to the test results in Figure 4, the MWCNT nanopaper had a sharp break at brittle fracture strength of about 1 MPa and strain 0.75%. The cured epoxy at first experienced strain hardening through plastic deformation and then a necking until fracture strain of 3.5% and tensile stress of 14 MPa was reached. On the other hand, the MWCNT nanopaper/epoxy composite strengthened until ultimate tensile stress of 24 MPa at fracture strain 2.9%.

**Figure 5:**
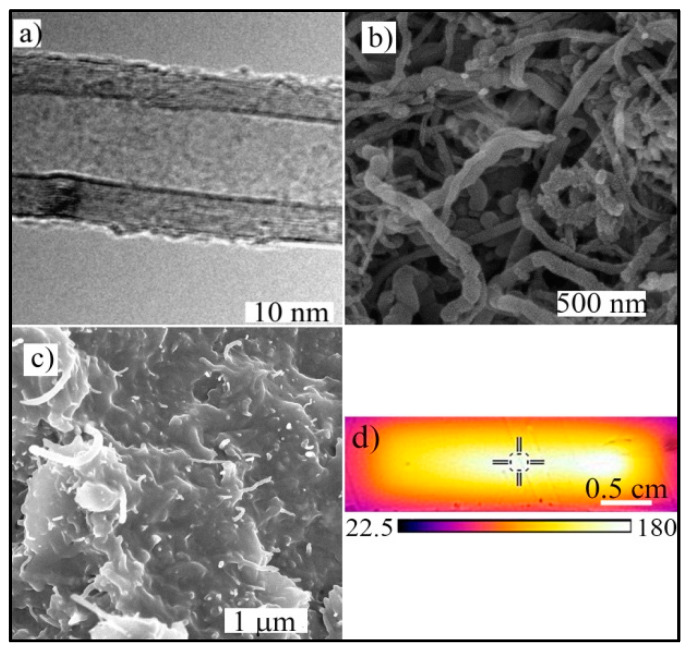
(**a**) A TEM image of an individual MWCNT with visible lines of rolled graphene layers at a distance of about 0.35 nm. (**b**) A SEM micrograph of the surface of the MWCNT nanopaper. (**c**) The cross-section of the MWCNT nanopaper/epoxy composite cured by Joule heating with cuts of individual MWCNTs, which protruded from the plane of the cross-section. (**d**) The thermographic image of the temperature distribution in the MWCNT nanopaper/epoxy strip in the course of curing at 4.6 V together with the temperature scale. The cross indicates the temperature of 173 °C.

A detailed view of the structure of an individual nanotube consisting of about 15 rolled sheets of graphene obtained by means of TEM is shown in Figure 5a. SEM micrographs depict the surface of a porous structure of the MWCNT nanopaper (Figure 5b), and the cross-section through the epoxy composite with the embedded conductive MWCNT nanopaper with some nanotubes protruding from the epoxy matrix surface together with cut nanotubes (bright spots on the surface), Figure 5c. Finally, Figure 5d depicts the temperature distribution of the MWCNT nanopaper/epoxy strip as measured by thermal camera Flir E5-XT in the course of the curing by means of the embedded Joule-heated carbon nanotubes. The temperature of the strip decreased towards the edges, apparently due to higher cooling in the marginal areas.

**Figure 6:**
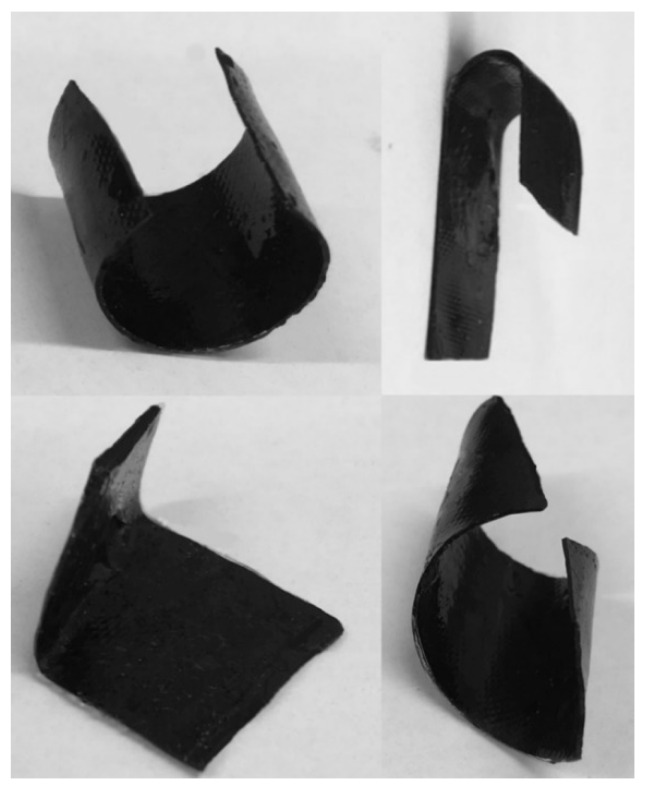
Different shapes of the MWCNT nanopaper/epoxy composites manipulated by hand after composite Joule heating to 70 °C and cooling below the *T*_g_ temperature of 53.9 °C to form new shapes.

The fabricated thermoset composites, which consisted of the epoxy matrix and the embedded conductive MWCNT nanopaper, offer a novel technique of formation by means of Joule heating. This self-heating, which was used for the curing of composite plates and strips, was repeated to quickly heat the composite plates (in 1–2 min) to the forming temperature of 70 °C, which was above their glass transition temperature *T*_g_ of 53.9 °C. At this temperature, the composites went from being rigid and glassy to rubbery and flexible, and thus highly formable. When a bending force was applied, the strips deformed easily to the chosen shapes, which they held after cooling below *T*_g_, despite the deformed embedded MWCNT nanopaper (Figure 6). 

**Figure 7:**
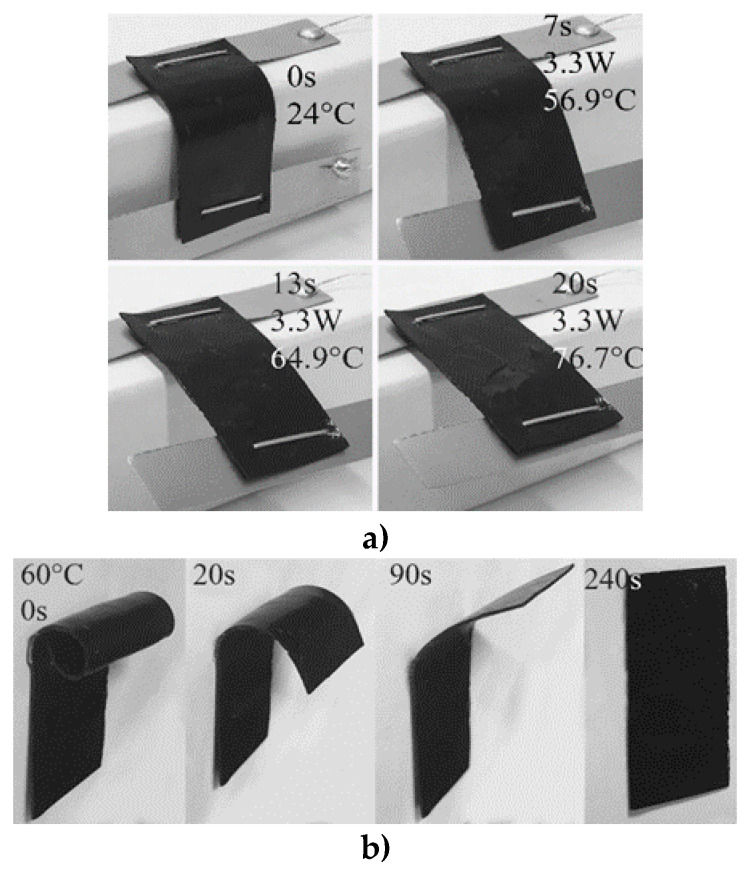
The time-dependent shape memory recovery of the MWCNT nanopaper/epoxy strip after (**a**) Joule heating to 76.7 °C (3.3 W), (**b**) heating in an oven to 60 °C. The respective temperatures, electric power, and times are included in the figure.

Surprisingly, when the bent strips were reheated (as described in Section 2), their original shape recovered. The photos illustrating recovery by means of both nanopaper self-heating and heating in an oven at 60 °C are shown in Figure 7a,b, respectively (see Supplementary Video). After the composite is heated over *T*_g_ to the transformation temperature of the shape memory, shape recovery stress in the embedded MWCNT nanopaper was released due to the epoxy matrix softening. Apparently, the embedded nanotubes were compressed on the inside surface of the bent strip and stretched on the outside. When the nanotubes were released and were able to move within the epoxy matrix, the deformed arrangement of the stretched portion and compressed portion of the nanopaper recovered to its initial arrangement. Moreover, the strip’s Joule heating through inter-nanotube contacts was more efficient than heating in an oven. Consequently, when Joule heating was applied to the bent composite strip attached to a box, the time of the strip shape’s unbending reached about 20 s (Figure 7a), which was about one order of magnitude shorter than that of the strip coiled portion’s recovery in the oven (Figure 7b).

**Figure 8:**
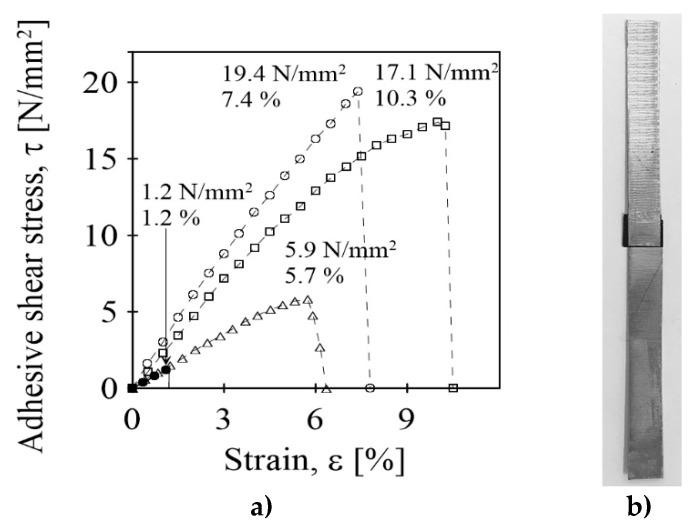
(**a**) The shear stress-strain relations for the bonded steel strips using the MWCNT nanopaper/epoxy composite (circles) and the epoxy adhesive (squares), and for the reheated adhesive bonding by the MWCNT nanopaper/epoxy composite at temperature of 55 °C (triangles) and 72 °C (filled circles). The respective ultimate stress and ultimate strain values are included in the figure. (**b**) The overlapped strips bonded by the MWCNT nanopaper/epoxy composite.

Rapid heating of an embedded nanopaper may facilitate polymerization of the epoxy components of the composite and thus aid its adhesive properties. To test this surmise, we used two zinc-coated steel strips (length 100 mm, width 10 mm, thickness 0.5 mm, overlap 10 mm, overlapping area 100 mm^2^) on which a few drops of the epoxy were deposited; the MWCNT nanopaper was attached, a few drops of the epoxy were added on top of the nanopaper surface, and then the second steel strip was overlaid. The overlapped strips were under pressure load of 300 kPa heated by electric power of 6 W to a temperature of about 160 °C for 5 min (Figure 8). In comparison, in the absence of the MWCNT nanopaper/epoxy composite, adhesive bonding of the identical zinc-coated steel strips with the ordinary epoxy resin under the same load of 300 kPa took 48 h: initially 24 h at room temperature, which was followed by curing at the bonding temperature of 60 °C in an oven for an additional 24 h. According to the results (Figure 8), the test of the adhesive strength of the bonding of the steel strips with both the MWCNT nanopaper/epoxy and epoxy adhesives gave ultimate shear stress values of 19.4 ± 0.4 N/mm^2^ and 17.1 ± 0.3 N/mm^2^, respectively. The corresponding ultimate strains were 7.4 and 10.3%. Thus, the MWCNT nanopaper/epoxy composite achieved comparable strength of bonding; yet, the required curing time was considerably shorter than when conventional epoxy adhesive was used. Moreover, the bonding retained its electrical conductivity and thus was able to be further modulated by the Joule heating. Once the temperature of the MWCNT nanopaper/epoxy composite, which glued together the steel strips, reached 72 °C within about 1 min, the bond of the steel strips was released at ultimate shear stress of 1.2 N/mm^2^. Hence, the bond of the steel strips, which overlapped at 100 mm^2^ and could withstand a total adhesive force of 1940 N (which is equivalent to about 200 kg of load under gravity) could be released by force of 120 N or about 12 kg of load under gravity, which can be readily exerted by human hand.

## 4. Discussion

We created an MWCNT nanopaper/epoxy composite consisting of electro-conductive MWCNT nanopaper, which was embedded in the epoxy matrix, and investigated its applications in several processing techniques. Previously, we showed that such an MWCNT nanopaper/epoxy composite can be used to monitor infiltration of epoxy resin through a glass fiber textile or a curing process of glass fiber/epoxy composite together with testing of its deformation [1]. In this paper, we focused on the process of epoxy resin curing by Joule heating. The results suggested that the composite was able to self-regulate the Joule heating (Figure 2). After initial rapid rise of the temperature of the cured prepolymer, a phase of decrease in temperature followed. It is probable that this decrease was due to volume changes resulting from polymerization. In the curing process, the volume of epoxy resin initially expands during polymerization [27]. As the curing continues, the volume shrinks. In this process, van der Walls interactions of the embedded nanotubes are apparently affected. This may change the nanopaper resistivity and/or number of inter-nanotube contacts, which in turn can be reflected in a reduction of applied power and a decrease in temperature. 

One of the advantages of the MWCNT/nanopaper composite was the facility of its formation by means of Joule heating. While heated, the composite quickly (within 1–2 min) reached *T*_g_ and became easily modellable into various shapes, which were retained once the composite cooled down. Such a utilization of Joule heating obviates the necessity of using an oven to form the given composites and thus may be beneficial e.g., for such processes done ad hoc, in restricted spaces or in situ in complex systems. 

The MWCNT nanopaper/epoxy composite could not only be easily formed, but also retained its shape memory. Namely, when Joule heating was reapplied to a molded MWCNT nanopaper/epoxy composite, it reverted to its original shape. Such a process was in an order of magnitude shorter when Joule heating was applied than when an oven was used. Thus, the shape recovery time as well as processability, light weight, and low manufacturing costs may be a highly desirable combination.

Facile forming and shape memory apart, another advantageous property of the MWCNT nanopaper/epoxy composite is its adhesiveness. Albeit no substantial difference in the strength of the bonding was observed when the composite was compared with a respective conventional epoxy adhesive, the time period necessary for curing and thus establishing a firm connection between the surfaces was substantially shorter in the nanomaterial, thanks to the possibility of application of Joule heating. In addition, repeated application of the DC current released these bonds, which suggests that materials based on the MWCNT nanopaper/epoxy composites might be used e.g., to form electrically controllable seams.

## 5. Conclusions

We combined a multi-walled carbon nanotube free-standing nanopaper and an epoxy resin into a reinforced thermosetting polymer matrix composite and demonstrated its versatile use in technological processes with advanced processing parameters, thus saving time and electrical energy. In our earlier papers [20,21,24], the nanopaper was introduced individually or embedded in thermoplastic polymers as a multifunctional sensor of ambient vapors or of a tensile and compressive stress. The nanopaper has been used as a built-in sensor in a glass fiber/epoxy composite to primarily measure composite deformation, as well to monitor epoxy resin infusion into layered glass fiber textiles and, subsequent curing process [1]. The nanopaper embedded in the epoxy matrix was investigated here as a thermosetting material in several practical applications listed in Table 1. However, at first, the properties and technological parameters of this conductive epoxy composite were measured to specify particulars of its manufacturing and use. In particular, we assessed the rise of nanopaper temperature in the course of its Joule heating at different voltages, temperature progression in the course of the MWCNT nanopaper/epoxy curing by Joule heating, TGA analysis of the MWCNT nanopaper/epoxy composite, tensile tests of the MWCNT nanopaper/epoxy composite, and shear stress-strain relations for the steel strips bonded by the MWCNT nanopaper/epoxy composite or conventional epoxy adhesive as well as for the reheated adhesive bonding by the MWCNT nanopaper/epoxy composite.

An innovative finding was self-regulation of the epoxy curing temperature (Figure 2). After the initial rapid rise in temperature of the cured prepolymer, the phase of decrease in temperature followed, probably due to volume changes resulting from the polymerization, as discussed in Section 4. The shape recovery measurement accentuated the contribution of Joule heating in the shape recovery process. This transition from deformed to undeformed arrangement was an order of magnitude shorter when Joule heating was applied, compared to when an oven was used for heating of the MWCNT nanopaper/epoxy composite, thanks to efficient heating through the inter-nanotube contacts. The second innovative finding was the possibility of the efficient and fast debonding of objects glued by the MWCNTnanopaper/epoxy composite. Reheating of the composite over T_g_ decreased its adhesive stress and consequent release was done readily by human hand. The list of possible applications of the MWCNT nanopaper/epoxy composite and modulation of its characteristics by Joule heating, as presented in Table 1, need not be exhaustive, as further feasible practical applications are being evaluated. 

## Figures and Tables

**Table 1:** Overview of introduced applications of the MWCNT nanopaper/epoxy composite.

Initial State of Material	Unit Operation	Particularity	Outcome
Nanopaper filled up with epoxy resin	Curing by Joule heating	Self-regulating Joule heating	MWCNTnanopaper/ epoxy composite
MWCNTnanopaper/ epoxy composite	Forming by Joule heating	Forming temperature reached in 1–2 min	Novel technique of forming
MWCNTnanopaper/ epoxy composite	Shape recovery by Joule heating	Recovery stress release by matrix softening	Short shape recovery time
Nanopaper filled up with epoxy resin	Adhesive bonding by Joule heating	Bonding time within minutes	Higher adhesion then by epoxy adhesive
MWCNTnanopaper/ epoxy composite	Debonding adhesive joint by Joule heating	Bond of steel strips released within 1 min	Easy release without destroying parts

## Supplementary Materials

The following are available online at https://www.mdpi.com/2073-4360/12/5/1030/s1.
